# Supplementary figures and images for: Effects of different separation methods on the physical and functional properties of extracellular vesicles
Source: PLoS One. 2020 Jul 7;15(7):e0235793. doi: 10.1371/journal.pone.0235793 (PMC7340315; doi:10.1371/journal.pone.0235793)

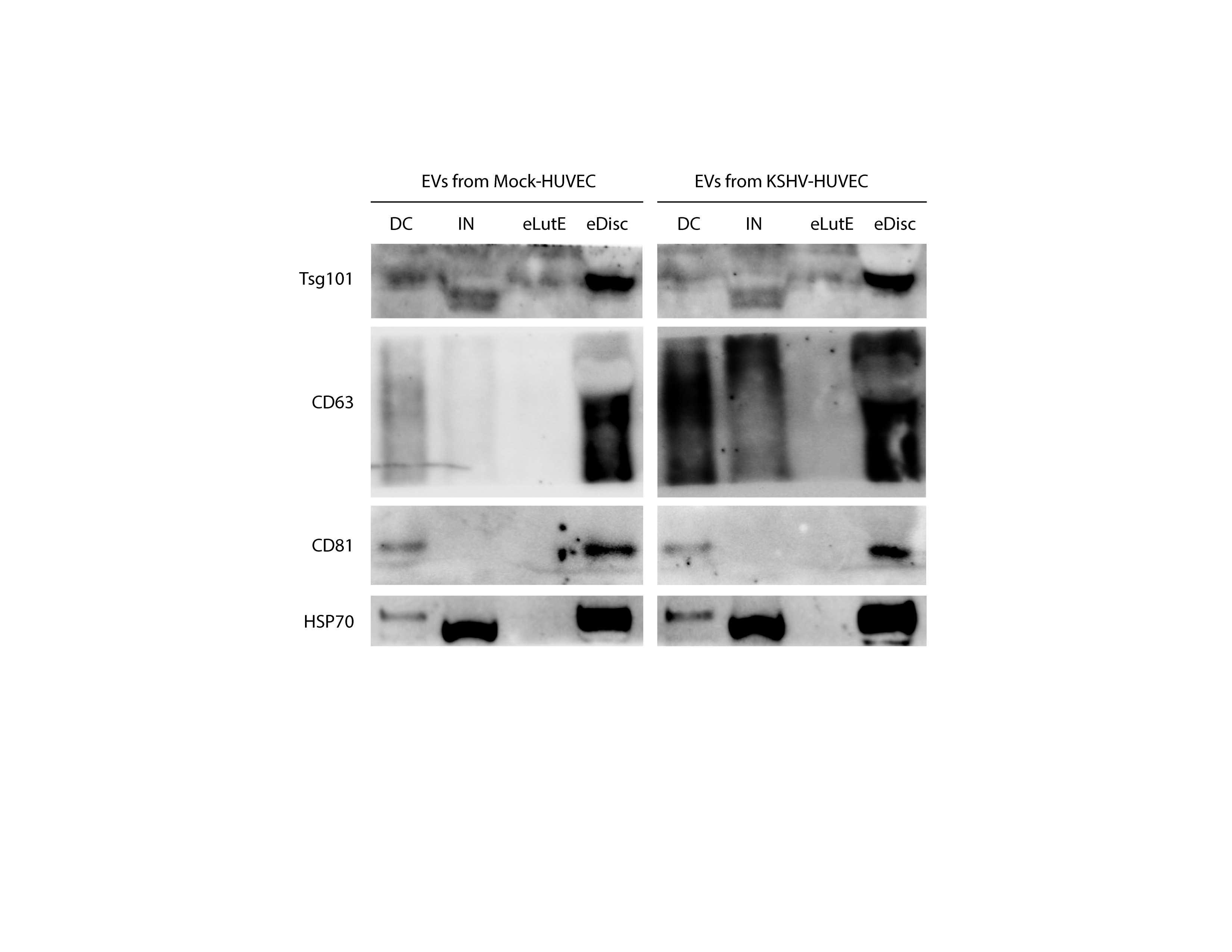

Supplement: S1 Fig — Two micrograms of protein lysate from each sample of EVs separated using the four different methods were loaded to the gel. The EV markers Tsg101, CD63, CD81, and HSP70 were analyzed by Western blotting. (TIF) [file pone.0235793.s001.tif]

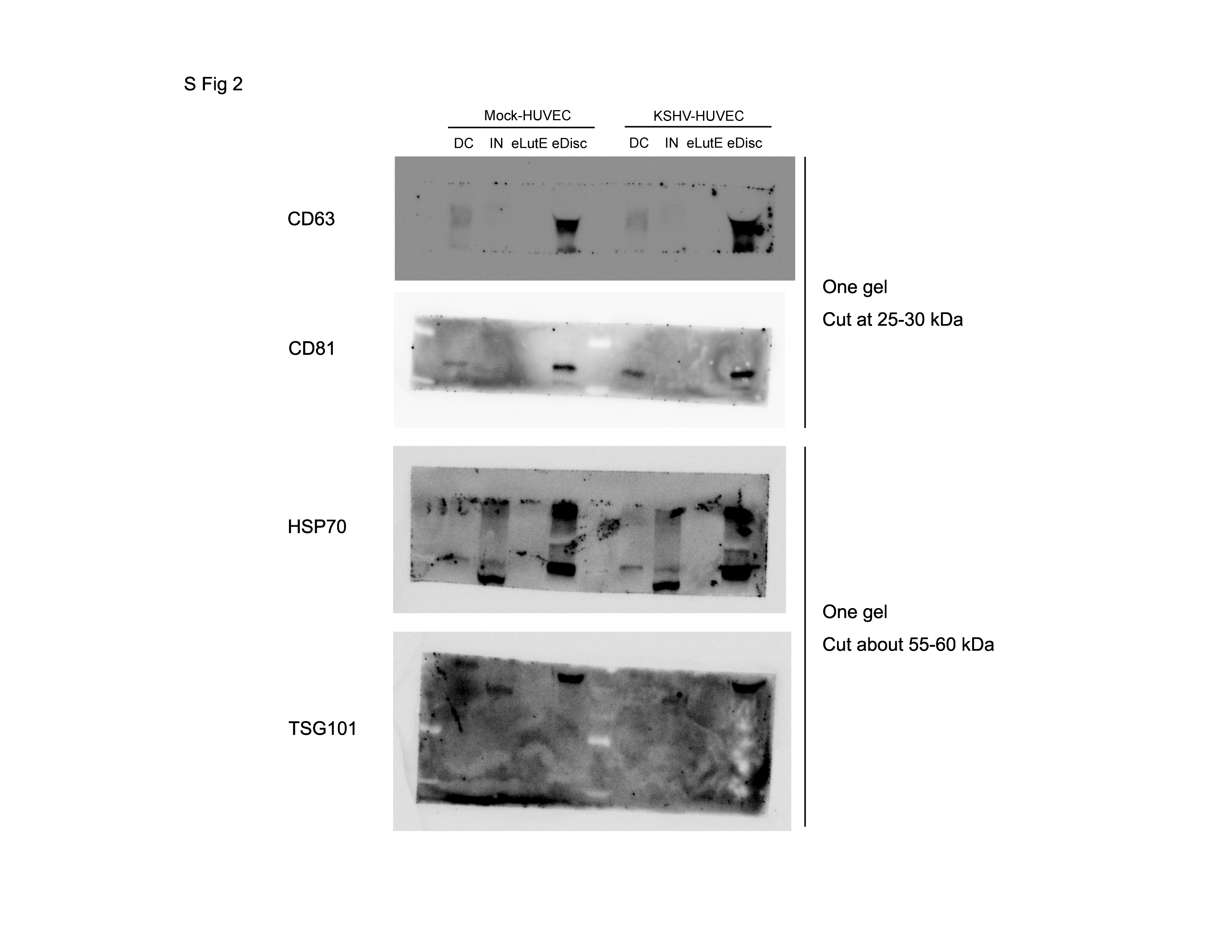

Supplement: S2 Fig — (TIF) [file pone.0235793.s002.tif]
